# Supplementary material for: Ovarian and Energy Status in Lame Dairy Cows at Puerperium and Their Responsiveness in Protocols for the Synchronization of Ovulation
Source: Animals (Basel). 2023 May 4;13(9):1537. doi: 10.3390/ani13091537 (PMC10177297; doi:10.3390/ani13091537)
Supplement: Supplementary file 1 [file animals-13-01537-s001.zip › animals-2358531-supplementary.pdf]

Table S1. Reproductive parameters and culling rate of control (group C) and lame cows with two presynchronization protocols (groups LC and LP).

Variable	Group					
	C	LC	LP	*p*-Value C vs LC vs LP	LC + LP	*p*-Value C vs LC + LP
n	27	26	26		52	
Non-cycling (%)	11.1 [a,1]	38.5[b]	34.6[b]	0.05	36.5[2]	0.02
Presynchronization success (%)	81.5	65.4	76.9	0.38	71.2	0.32
Synchronization success (%)	92.6	84.6	92.3	0.65	88.5	0.71
Ovulation (%)	81.5	80.8	76.9	0.91	78.9	0.78
Non-cycling Ovulated (%)	100	70	77.7	0.55	73.7	0.44
FSCR (%)	37.0	26.9	16.0	0.23	21.6	0.14
Pregnant by 200d (%)	88.9	70.8	87.5	0.18	79.2	0.29
AIs/pregnancy (mean ± SE)	3.1 ± 0.4	2.8 ± 0.5	3.0 ± 0.5	0.87	2.9 ± 0.3	0.75
Days open (median)	123	114	111	0.95	112	0.94
Culled (%)	0 [a,1]	23.1[b]	19.2 [b]	0.03	21.2 [2]	0.01

[a,b] Different letters denote statistical differences between the groups Control, Lame Control and Lame Progesterone ($p < 0.05$);

[1,2] Different numbers denote statistical differences between the groups Control and all Lame (LC + LP) ($p < 0.05$)

Table S2. Reproductive parameters and culling rate of cows with elevated ß-hydroxybutyric acid (BHBA+) and non-esterified fatty acids (NEFA+) during the study period.

Variable	Group					
	**BHBA-**	**BHBA+**	***p*-Value BHBA**	**NEFA-**	**NEFA+**	***p*-Value NEFA**
n	72	7		43	36	
Non-cycling (%)	27.8	28.6	0.96	27.9	27.8	0.99
Presynchronization success (%)	73.6	85.7	0.48	72.1	77.8	0.56
Synchronization success (%)	90.3	85.7	0.70	88.4	91.7	0.63
Ovulation (%)	79.2	85.7	0.68	79.1	80.6	0.87
Non-cycling Ovulated (%)	80.0	50.0	0.41	83.3	70.0	0.62
FSCR (%)	26.8	28.6	0.91	27.9	25.7	0.83
Pregnant by 200d (%)	83.8	71.4	0.60	82.9	82.4	0.95
AIs/pregnancy (mean ± SE)	2.9 ± 0.3	3.6 ± 1.1	0.53	3.2 ± 0.4	2.7 ± 0.4	0.44
Days open (median)	112	134	0.40	115	109	0.76
Culled (%)	15.3	0	0.58	14.0	13.9	0.99

Table S3. Reproductive parameters and culling rate of cows with moderate or marked lameness and with lesions due to claw horn disruptions (CHDL) or infectious diseases (ID).

	Group			Group		
**Variable**	**MODERATE**	**MARKED**	***p*-Value**	**CHDL**	**ID**	***p*-Value**
n	16	36		37	15	
Non-cycling (%)	25.0	41.7	0.25	40.5	26.7	0.35
Presynchronization success (%)	75.0	69.4	0.68	64.9	86.7	0.12
Synchronization success (%)	93.8	86.1	0.65	83.8	100	0.10
Ovulation (%)	81.3	77.8	1.0	73.0	93.3	0.10
Non-cycling Ovulated (%)	75.0	73.3	1.0	66.7	100.0	0.53
FSCR (%)	25.0	20.0	0.72	13.9 [1]	40.0 [2]	0.04
Pregnant by 200d (%)	81.3	78.1	1.0	73.5	92.9	0.13
AIs/pregnancy (mean ± SE)	3.0 ± 0.5	2.8 ± 0.5	0.38	3.0 ± 0.4	2.6 ± 0.6	0.40
Days open (median)	115	111	0.84	114	110	0.33
Culled (%)	0 [a]	30.6 [b]	0.01	21.6	20.0	0.90

[a, b] Different letters denote statistical differences between the groups of moderate and severe lameness ($p < 0.05$)

[1, 2] Different numbers denote statistical differences between the groups with Claw Horn Disruption Lesions (CHDL) and Infectious Diseases (ID) ($p < 0.05$)
